# IL-27 and TGFβ mediated expansion of Th1 and adaptive regulatory T cells expressing IL-10 correlates with bacterial burden and disease severity in pulmonary tuberculosis

**DOI:** 10.1002/iid3.68

**Published:** 2015-06-18

**Authors:** Nathella P Kumar, Kadar Moideen, Vaithilingam V Banurekha, Dina Nair, Rathinam Sridhar, Thomas B Nutman, Subash Babu

**Affiliations:** 1 National Institutes of Health—International Center for Excellence in ResearchChennai, India; 2 National Institute for Research in TuberculosisChennai, India; 3 Government Stanley Medical HospitalChennai, India; 4 Laboratory of Parasitic Diseases, National Institutes of Allergy and Infectious Diseases, National Institutes of HealthBethesda, Maryland, USA

**Keywords:** Cytokines, IL-10, regulation, T cells, tuberculosis

## Abstract

CD4^+^ T cell expression of IL-10 is an important mechanism controlling immunity to tuberculosis (TB). To identify the CD4^+^ T cell subsets producing IL-10 in human TB, we enumerated the frequencies of IL-10 expressing CD4^+^ T cell subsets following TB—antigen stimulation of cells from individuals with pulmonary (PTB) and latent TB (LTB). We first demonstrate that TB antigens induce an expansion of IL-10 expressing Th1 (IL-10^+^, IFNγ^+^, T-bet^+^), Th2 (IL-10^+^, IL-4^+^, GATA-3^+^), Th9 (IL-10^+^, IL-9^+^, IL-4^−^), Th17 (IL-10^+^, IL-17^+^, IFNγ^−^), and natural and adaptive regulatory T cells [nTregs; IL-10^+^, CD4^+^, CD25^+^, Foxp3^+^ and aTregs; IL-10 single^+^, CD4^+^, CD25^−^, Foxp3^−^] in PTB and LTB individuals, with frequencies being significantly higher in the former. However, only Th1 cells and adaptive Tregs expressing IL-10 exhibit a positive relationship with bacterial burdens and extent of disease in PTB. Finally, we show that IL-27 and TGFβ play an important role in the regulation of IL-10^+^ Th cell subsets. Thus, active PTB is characterized by an IL-27 and TGFβ mediated expansion of IL-10 expressing CD4^+^ T cell subsets, with IL-10^+^ Th1 and IL-10^+^ aTreg cells playing a potentially pivotal role in the pathogenesis of active disease.

## Introduction

IL-10 is a regulatory cytokine with a broad spectrum of activities, predominantly anti-inflammatory, and immuno-suppressive 1,2. IL-10 is known to downregulate the immune response in a variety of settings 3. Thus, during acute infections, IL-10 is essential in limiting host tissue damage as a consequence of excessive inflammation 1,2,4. In contrast, various pathogens exploit the suppressive activity of IL-10 to evade the immune system and establish persistent infections 5. IL-10 is produced by virtually all innate and adaptive immune cells, including dendritic cells, macrophages, B cells, CD4^+^, and CD8^+^ T cells 2,4. However, it has recently become clear that effector CD4^+^ T cell production of IL-10 plays an important role in limiting host pathology and promoting chronicity of infection 6. Effector function in CD4^+^ T cells is mediated by four major subsets: Th1, Th2, Th9, and Th17 7. In addition, regulatory T cells, both innate (nTregs) and adaptive (aTregs), are also known to be major producers of IL-10 in immunity to infections 8.

IL-10 plays a very important role in the regulation of host immune response against *Mycobacterium tuberculosis* infection 9. IL-10 is known to cause inhibition of macrophage effector functions, with reduced bacterial killing and impaired cytokine/chemokine secretion 10,11, block the chemotactic factors that control dendritic cell trafficking to the lymph nodes 12, dampen the differentiation of naive CD4^+^ T cells to Th1 cells 13 and finally suppress Th1, Th2, and Th17 cytokine production 14,15. IL-10 is increased in individuals with active TB and a higher capacity to produce IL-10 is associated with an increase in the disease incidence 9. Moreover, IL-10 production is higher in anergic patients, suggesting the TB induced IL-10 production can suppress an effective immune response 16.

Although, IL-10 plays such a significant role in the immune response to TB, the cellular origins of IL-10 from CD4^+^ T cells is still not clear in TB infection and disease. By using multi-parameter flow cytometry to examine IL-10 expression in active pulmonary TB (PTB) and latent TB (LTB) individuals, we demonstrate that PTB is associated with expanded IL-10 expression by all CD4^+^ helper T cell subsets following TB antigen stimulation and that IL-10 expressing Th1 cells and aTregs exhibit the highest degree of correlation with bacterial burden and lung pathology. Finally, we demonstrate that IL-27 and TGFβ are major regulators of IL-10 expression in CD4^+^ T cells.

## Results

### Th1, Th2, Th9, Th17, and Tregs express IL-10 in active TB

To identify the expression pattern of IL-10 in effector and regulatory CD4^+^ T cells, we examined the expression of IL-10 in CD4^+^ T cells expressing IFNγ (Th1), IL-4 (Th2), IL-9 (Th9), IL-17 (Th17), CD25^+^ Foxp3^+^ (nTregs), and CD25^-^Foxp3^−^ (aTregs) in active and latent TB individuals. The gating strategy for CD4^+^ T cells from a representative active TB individual is shown in Figure S1A. As shown in Figure S1B, we demonstrate using multi-parameter flow cytometry that Th1, Th2, Th9, Th17, and Treg cells co-express IL-10. In addition, we also used multi-color intracellular staining to show that Th9 cells that co-express IL-10 do not express IL-4 and that Th17 cells that co-express IL-17 do not express IFNγ (data not shown). Finally, we also demonstrate that Th1 cells that express IFNγ and IL-10, also express T-bet, while Th2 cells that express IL-4 and IL-10, also express GATA-3 (Figure S1C). Thus, both effector and regulatory CD4^+^ T cells can co-express IL-10 in active TB.

### Expansion of IL-10 expressing Th1, Th2, Th9, Th17, and Tregs in response to TB antigens in active and latent TB

**Figure 1:**
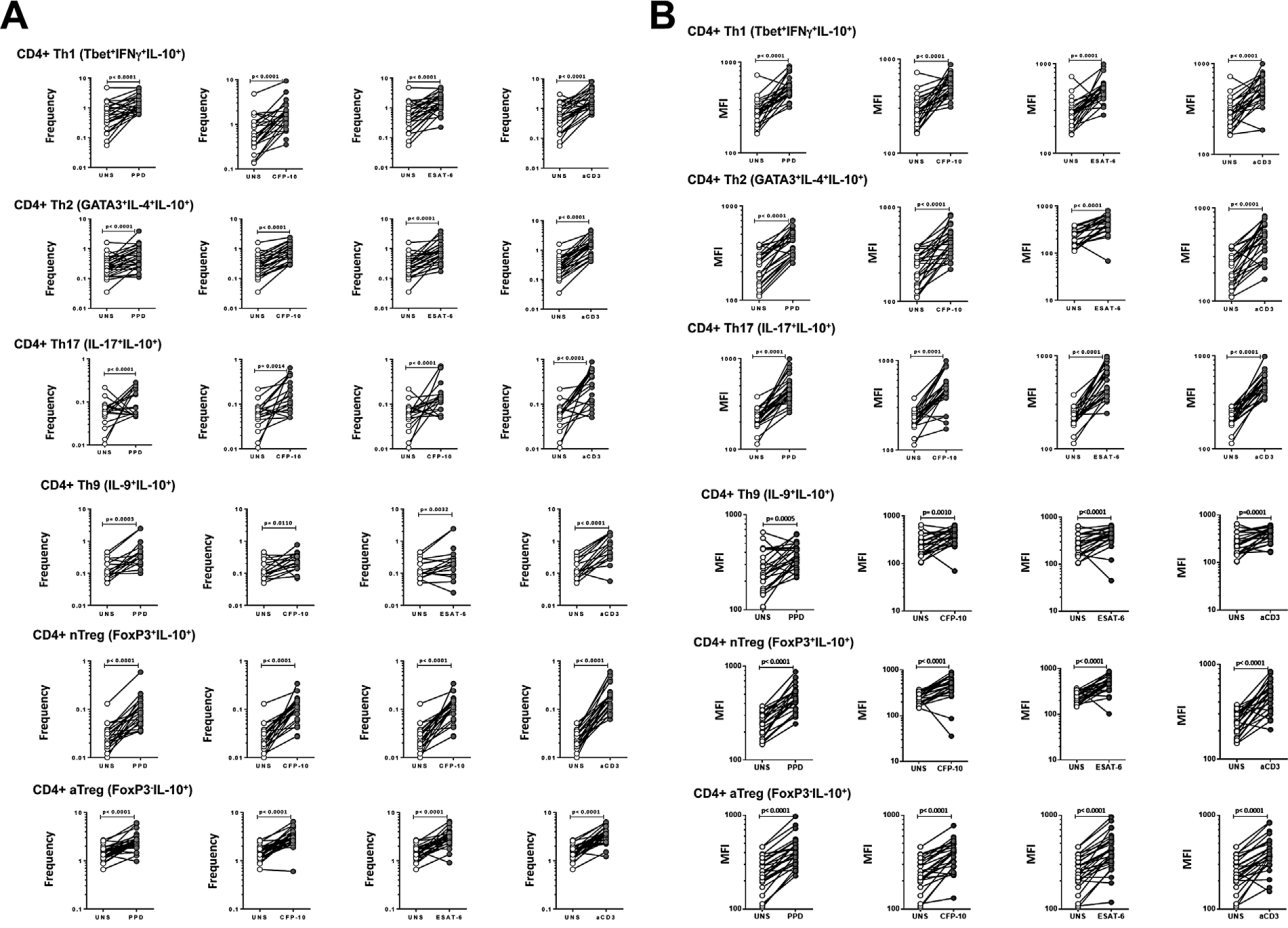
Expansion of IL-10 expressing CD4^+^ T cell subsets in response to TB antigens and anti-CD3 in PTB. (A) Whole blood from PTB individuals (*n* = 30) was stimulated with PPD, CFP-10, ESAT-6, or anti-CD3 for 24 and the frequencies of IL-10^+^ Th1 cells (IL-10^+^ IFNγ^+^ T-bet^+^), IL-10^+^ Th2 cells (IL-10^+^ IL-4^+^ GATA-3^+^), IL-10^+^ Th9 cells (IL-10^+^ IL-9^+^ IL-4^−^), IL-10^+^ Th17 cells (IL-10^+^ IL-17^+^ IFNγ^−^), IL-10^+^ nTregs (IL-10^+^ CD25^+^ Foxp3^+^), and IL-10^+^ aTregs (IL-10^+^ CD25^−^ Foxp3^−^) were estimated by flow cytometry. (B) The geometric mean fluorescence intensity of IL-10 expression on each of the above CD4^+^ T cell subsets was estimated by flow cytometry. Results are shown as line diagrams with each line representing a single individual. *P* values were calculated using the Wilcoxon signed rank test.

To determine the frequency of antigen-responsive effector and regulatory CD4^+^ T cells expressing IL-10, we stimulated whole blood with PPD or ESAT-6 or CFP-10 or anti-CD3 for 24 h and measured the frequencies of CD4^+^ T cells expressing IL-10 in PTB (*n* = 30) individuals. As shown in Figure 1A, PTB individuals exhibited significantly enhanced frequencies of TB antigen and anti-CD3-stimulated Th1 (IFNγ^+^, T-bet^+^), Th2 (IL-4^+^, GATA-3^+^), Th9 (IL-9^+^, IL-4^-^), Th17 (IL-17^+^, IFNγ^−^), nTreg (CD25^+^, Foxp3^+^), and aTreg (CD25^−^, Foxp3^−^) cells co-expressing IL-10. This expansion was observed with all the TB antigens used including PPD, ESAT-6, and CFP-10, or with anti-CD3. PTB individuals also exhibited an increased per cell magnitude of IL-10 expression (as assessed by MFI) on the different CD4^+^ T cell subsets upon stimulation with TB antigens as well as anti-CD3 (Fig. 1B). Thus, active pulmonary TB appears to be characterized by an antigen-responsive expansion of IL-10^+^ CD4^+^ T cells that also are producing more IL-10 per cell. In addition, we also examined the antigen-specific expansion of CD4^+^ T cells subsets expressing IL-10 in latent TB individuals. As shown in Figure S2, latent TB was also characterized by an antigen-specific expansion of CD4^+^ T cell subsets expressing IL-10.

### Increased TB-antigen induced frequencies of Th1, Th2, Th17, and Treg cells expressing IL-10 in active TB

**Figure 2:**
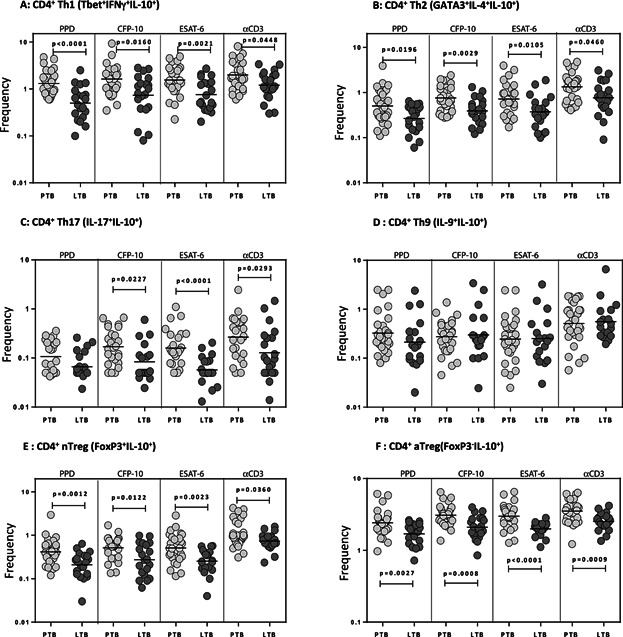
Increased antigen-specific frequencies of IL-10 expressing CD4^+^ T cell subsets in PTB. Whole blood from PTB (*n* = 30) and LTB (*n* = 20) individuals was stimulated with PPD, CFP-10, ESAT-6, and anti-CD3 for 24 h, and the frequencies of (A) IL-10^+^ Th1 cells (IL-10^+^ IFNγ^+^ T-bet^+^), (B) IL-10^+^ Th2 cells (IL-10^+^ IL-4^+^ GATA-3^+^), (C) IL-10^+^ Th17 cells (IL-10^+^ IL-17^+^ IFNγ^-^), (D) IL-10^+^ Th9 cells (IL-10^+^ IL-9^+^ IL-4^−^), (E) IL-10^+^ nTregs (IL-10^+^ CD25^+^ Foxp3^+^), and (F) IL-10^+^ aTregs (IL-10^+^ CD25^−^ Foxp3^−^) were estimated by flow cytometry. Stimulated frequencies are shown as scatter plots with the line representing the geometric means. Frequencies following stimulation with antigens are depicted as net frequencies (with baseline values subtracted). *P* values were calculated using the Mann–Whitney *U* test.

To compare the frequencies of TB antigen induced IL-10^+^ effector and regulatory CD4^+^ T cells in active and latent TB, we measured the frequencies of Th1, Th2, Th9, Th17, and Treg cells expressing IL-10 in PTB (*n* = 30) and LTB (*n* = 20) individuals. As shown in Figure 2A, we observed significantly increased frequencies of Th1 cells expressing IL-10 (IFNγ^+^, T-bet^+^, IL-10^+^) following stimulation with PPD, ESAT-6, CFP-10, and anti-CD3 in PTB compared to LTB individuals. As shown in Figure 2B, we also observed significantly increased frequencies of Th2 cells expressing IL-10 (IL-4^+^, GATA-3^+^, IL-10^+^) following stimulation with PPD, ESAT-6, CFP-10, and anti-CD3 in PTB compared to LTB individuals. Similarly, we also observed significantly increased frequencies of Th17 cells expressing IL-10 (IL-17^+^, IL-10^+^, IFNγ^−^) following stimulation with ESAT-6, CFP-10, and anti-CD3 but not with PPD (Fig. 2C). In contrast, we did not observe any significant difference in the frequencies of Th9 cells expressing IL-10 (IL-4^+^, IL-10^+^, IL-4^−^) following stimulation with PPD, ESAT-6, CFP-10, and anti-CD3 between PTB and LTB individuals (Fig. 2D). Finally, we observed increased frequencies of IL-10^+^ nTregs (CD25^+^, Foxp3^+^, IL-10^+^) and IL-10 single^+^ aTregs (CD25^−^, Foxp3^−^, IL-10^+^) in response to TB antigens and anti-CD3 in PTB compared to LTB individuals (Fig. 2E and F). Thus, active TB is characterized by an expanded frequencies of IL-10 producing CD4^+^ T cell subsets following antigen stimulation.

### Frequencies of Th1 cells and aTregs expressing IL-10 exhibit a positive relationship with bacterial burden and extent of disease

**Figure 3:**
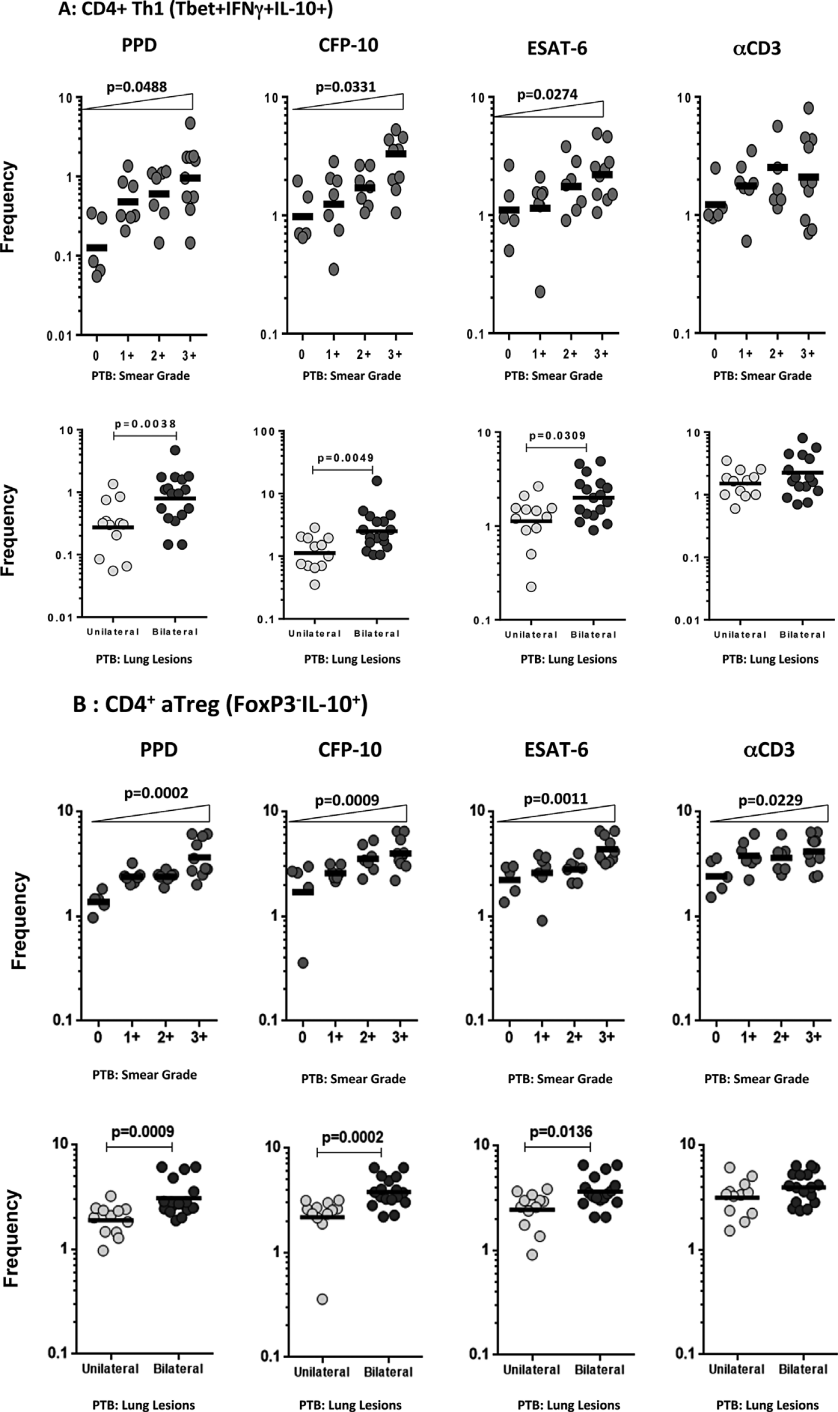
Positive relationship between IL-10^+^ Th1 and aTreg cell frequencies and bacterial burdens/disease extent in active PTB. (A) The relationship between the frequencies of IL-10^+^ Th1 cells following TB antigen or anti-CD3 stimulation and the smear grades are shown as scatter plots in the upper panel. (B) The relationship between the frequencies of IL-10^+^ aTregs cells following TB antigen or anti-CD3 stimulation and the smear grades are shown as scatter plots in the upper panel. The frequencies of IL-10^+^ aTregs cells in unilateral disease versus bilateral disease is also shown as scatter plots in the bottom panel. *P* values were calculated using the one way ANOVA Posttest for linear trends. The frequencies of IL-10^+^ Th1 cells in unilateral disease versus bilateral disease is also shown as scatter plots in the bottom panel. *P* values were calculated using the Mann–Whitney *U* test.

To assess the role of IL-10 expression in altering bacterial burden/extent of disease, we examined the relationship between the frequencies of Th1, Th2, Th9, Th17, and Treg cells expressing IL-10 in PTB individuals and either smear grades or extent of disease (unilateral vs. bilateral). As can be shown in Figure 3A, the antigen-driven frequencies of IL-10^+^ Th1 cells exhibited a positive relationship with increasing bacterial burdens as determined by the smear grades by culture/microscopy. Similarly, the TB- antigen stimulated frequencies of IL-10^+^ Th1 cells were also significantly higher in PTB individuals with bilateral disease compared to those with unilateral disease. Similarly, as shown in Figure 3B, the antigen (PPD, ESAT-6, or CFP-10)-stimulated frequencies of IL-10^+^ aTreg cells exhibited a positive relationship with increasing bacterial burdens and were also significantly higher in PTB individuals with bilateral disease compared to those with unilateral disease. In contrast, there was no significant relationship between the frequencies of IL-10^+^ Th2 cells, IL-10^+^ nTregs, IL-10^+^ Th9 cells, or IL-10^+^ Th17 cells with bacterial burdens or extent of disease in PTB individuals (data not shown). Thus, both Th1 and aTreg cells expressing IL-10 appear to be associated with the degree of pathology in active pulmonary TB.

### IL-27 and TGFβ regulate the antigen-stimulated frequencies of IL-10 expressing CD4^+^ T cell subsets in PTB

**Figure 4:**
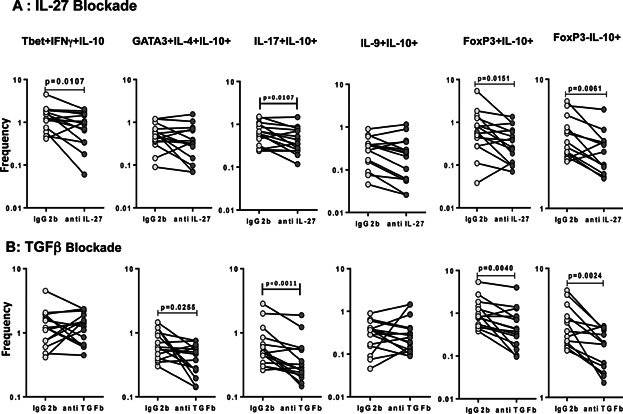
Neutralization of IL-27 and TGFβ significantly diminishes the frequencies of IL-10^+^ CD4^+^ T cell subsets in PTB. Whole blood from PTB individuals (*n* = 15) was stimulated with PPD in the presence of anti-IL-27 Ab (A) or anti-TGFβ Ab (B) or isotype control antibodies and the frequencies of IL-10^+^ Th1 cells (IL-10^+^ IFNγ^+^ T-bet^+^), IL-10^+^ Th2 cells (IL-10^+^ IL-4^+^ GATA-3^+^), IL-10^+^ Th9 cells (IL-10^+^ IL-9^+^ IL-4^−^), IL-10^+^ Th17 cells (IL-10^+^ IL-17^+^ IFNγ^-^), IL-10^+^ nTregs (IL-10^+^ CD25^+^ Foxp3^+^), and IL-10^+^ aTregs (IL-10^+^ CD25^−^ Foxp3^−^) were estimated by flow cytometry. Results are shown as line diagrams with each line representing a single individual. *P* values were calculated using the Wilcoxon signed rank test.

Since IL-27 and TGFβ are known to regulate IL-10 expression in CD4^+^ T cells 17,18, we sought to determine the role of these regulatory cytokines in the regulation of IL-10 expressing CD4^+^ T cell subsets in active TB. Thus, we stimulated whole blood from a subset of PTB individuals (*n* = 15) with PPD in the presence of neutralizing antibodies to IL-27 or TGFβ and measured the frequencies of IL-10^+^ CD4^+^ T cells (Fig. 4). As shown in Figure 4A, blockade of IL-27 resulted in a significant decrease in the frequencies of PPD-stimulated IL-10^+^ Th1 cells, IL-10^+^ Th17 cells and IL-10^+^ nTregs and IL-10^+^ aTregs but not IL-10^+^ Th2 cells and IL-10^+^ Th9 cells. In contrast, as shown in Figure 4B, TGFβ blockade resulted in a significant decrease in the frequencies of PPD-stimulated IL-10^+^ Th2 cells, IL-10^+^ Th17 cells, IL-10^+^ nTregs, and IL-10^+^ aTregs but not IL-10^+^ Th1 cells and IL-10^+^ Th9 cells.

### CTLA-4 and PD-1 minimally regulate the antigen-stimulated frequencies of IL-10 expressing CD4^+^ T cell subsets in PTB

**Figure 5:**
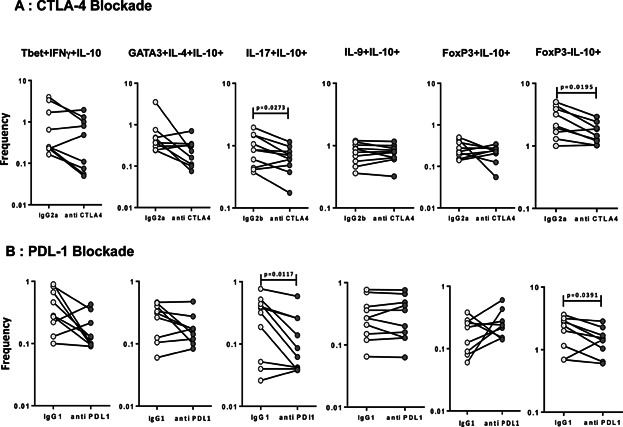
Blockade of CTLA-4 and PDL-1 minimally affects the frequencies IL-10^+^ CD4^+^ T cells in PTB. Whole blood from PTB individuals (*n* = 9) was stimulated with PPD (10 μg/ml) in the presence of anti- CTLA-Ig (A) or anti-PDL-1 (B) or isotype control antibody for 24 h and the frequencies of IL-10^+^ Th1 cells (IL-10^+^ IFNγ^+^ T-bet^+^), IL-10^+^ Th2 cells (IL-10^+^ IL-4^+^ GATA-3^+^), IL-10^+^ Th9 cells (IL-10^+^ IL-9^+^ IL-4^−^), IL-10^+^ Th17 cells (IL-10^+^ IL-17^+^ IFNγ^−^), IL-10^+^ nTregs (IL-10^+^ CD25^+^ Foxp3^+^), and IL-10^+^ aTregs (IL-10^+^ CD25^−^ Foxp3^−^) were estimated by flow cytometry. Results are shown as line diagrams with each line representing a single individual. *P* values were calculated using the Wilcoxon signed rank test.

Because CLTA-4 and PD-1 are also known regulators of IL-10 expressing in CD4^+^ T cells 19,20, we also wanted to determine the role of these receptors in the in the regulation of IL-10 expressing CD4^+^ T cell subsets in active TB. Thus, we stimulated whole blood from PTB individuals (*n* = 9) with PPD in the presence of blocking antibodies to CTLA-4 or PDL-1 and measured the frequencies of IL-10 expressing CD4^+^ T cell subsets (Fig. 5). As shown, blockade of CTLA-4 and PD-1 signaling resulted in a significant dimunition in the PPD-stimulated frequencies of IL-10^+^ Th17 cells and IL-10^+^ aTregs but not the other CD4^+^ T cell subsets expressing IL-10. Thus the co-inhibitory molecules—CTLA-4 and PD-1 appear to play only a minimal role in the regulation of IL-10 expressing CD4^+^ T cell subsets in active TB.

## Discussion

Although there is now considerable information regarding the regulatory effects of IL-10 on immune responses and pathology, there is much less known about the cellular sources of the cytokine and the specific signals that govern its induction. Although myeloid cells can contribute to IL-10 production, it appears that T cells are the major source of IL-10 in a variety of infections 6. Traditionally, IL-10 from T cells was shown to be mainly produced by the Th2 subset. However in addition to Th2 cells, a number of different CD4^+^ T cell subsets are now known to produce IL-10 as part of their effector function. These include natural as well as adaptive Tregs, Th1, Th9, and Th17 cells 6. Indeed, while aTregs (Foxp3^−^ Tregs) are known to be the major producers of IL-10 in filarial infections 21 and visceral leishmaniasis 22, Th1 cells are an important source of IL-10 in others, including malarial, infections 23, and in animal models of toxoplasmosis 24 and cutaneous leishmaniasis 25. IL-10 producing Th9 cells have also been shown to play an important role in immunity against intestinal helminth infections 26. Finally, IL-10 producing Th17 cells are important components of the immune response against bacterial infections, such as *Staphyloccus aureus* 27. In contrast, IL-10 production from CD4^+^ CD25^+^ Tregs is known to control *Leishmania major* persistence and immunity 28. Thus, different infections elicit different CD4^+^ T cells producing IL-10.

Our study identifies the effector and regulatory CD4^+^ T cell populations that express IL-10 in active and latent TB individuals. Our data first demonstrate that IL-10 production is not confined to a particular CD4^+^ T cell subset but is in fact expressed in all the different T cell subsets examined. Thus, Th1 cells, characterized by co-expression of IFNγ and T-bet; Th2 cells, characterized by co-expression of IL-4 and GATA-3; Th9 cells, characterized by co-expression of IL-9 and lack of IL-4; Th17 cells, characterized by co-expression of IL-17 and lack of IFNγ; nTregs, characterized by co-expression of CD25 and Foxp3 and aTregs, characterized by the lack of CD25 and Foxp3 expression can all express IL-10 both spontaneously and following TB-antigen stimulation in active and latent TB individuals. Moreover, these CD4^+^ T cell subsets all exhibit expansion following TB-antigen stimulation in short-term cultures suggesting that these IL-10 producing CD4^+^ T cells are antigen-responsive. In addition, our data also reveal that the per cell production of IL-10 in each of these CD4^+^ T cells subsets is significantly enhanced upon TB-antigen stimulation. Thus, TB disease appears to reflect an increase in IL-10 expression by CD4^+^ T cells quantitatively and qualitatively. This expansion is not confined to double cytokine expressing T cells since single producers of IL-10 are also expanded in each group. In addition, this expansion of IL-10^+^ CD4^+^ T cells is not confined to active TB individuals alone since a moderate expansion of these cells is also observed in latent TB individuals. Of greater interest, however is the finding that IL-10 expression was significantly higher in active TB compared to latent TB. Whether the presence of elevated frequencies of IL-10^+^ CD4^+^ T cells in PTB also translates to increased responses to bystander or third party antigens remains to be determined.

Since IL-10 is known to be a major cytokine in the immune response to TB 9,29, it is not surprising to find that most of the effector T cell subsets examined exhibit the ability to produce IL-10. The induction of IL-10 by the host probably is a double-edged sword with both host protective and host deleterious properties. Since IL-10 is known to limit tissue pathology 3, IL-10 can play a beneficial role in the immune response. Conversely, IL-10 is also known to promote pathogen persistence by limiting sterilizing immunity 6. To investigate which of the possibilities is operational in the setting of active TB, we examined the relationship of Th1, Th2, Th9, Th17, and Treg expressing IL-10 to the bacterial burdens (as measured by smear grades using microscopy). In so doing, we found a striking positive association between IL-10^+^ Th1 and IL-10^+^ aTreg cells and the smear grades indicating that as the frequency of IL-10^+^ Th1 and aTreg cells increases so do the bacterial burdens in the lung. While we cannot prove causation nor provide evidence on which occurs first, we nevertheless provide an important link between the pathogenesis of infection/disease and IL-10 production by Th1 and aTreg cells. In addition, it is certainly plausible that the increased expansion of IL-10^+^ CD4^+^ T cells in PTB individuals is merely a reflection of a more exuberant immune response encompassing IL-10 producing CD4^+^ T cell subsets. Thus, the enhanced frequencies of IL-10^+^ Th1 cells in *M. tuberculosis* infection differs from that observed in Toxoplasma or Leishmania, wherein the T cells serve to limit pathology 24,25 and, similar to malaria infections, the T cells probably abrogate immunity 23. Our study also identifies a novel but important role for Foxp3^−^ Tregs as a major source of IL-10, potentially influencing the progression of TB pathogenesis. As further confirmation for a host deleterious association for IL-10^+^ Th1 and aTreg cells in active TB, we also observed significantly higher frequencies of these cells in bilateral disease compared to unilateral disease. Since, individuals with unilateral disease have less extensive pathology compared to those with bilateral disease, this suggests that IL-10^+^ Th1 and aTreg cells are also associated with increased lung pathology in active TB. Our study also provides some interesting data on the regulation of CD4^+^ T cell subsets in active TB independent of IL-10 expression. Not only were we able to show that Th9 cells, although antigen responsive, play little role in active (vs. latent) TB but we were also able to demonstrate that Th17 cells do expand in response to TB antigens, a finding reminiscent of responses to other bacterial pathogens 30.

Several factors, including cytokines and cell surface receptors—for example, IL-27 17, IL-12 31, TGFβ 18, Type 1 IFN 32, CTLA-4 19, PD-1 20, and the Notch pathway 33 induce IL-10 from effector or regulatory T cells. The role of these factors in regulating IL-10 secretion from CD4^+^ T cells in TB is not well understood. Our study reveals novel roles for IL-27 and TGFβ in the regulation of IL-10 production from CD4^+^ T cells. Thus, we demonstrate differential requirements for exogenous cytokines in stimulating IL-10 expression in different CD4^+^ T cell subsets. For example, IL-10 expression in Th1 cells is dependent on IL-27 but not TGFβ; IL-10 expression in Th2 cells is dependent on TGFβ but not IL-27 and IL-10 expression in Th17, nTreg, and aTreg cells is dependent on both IL-17 and TGFβ. In contrast, IL-10 expression in Th9 cells is not dependent on either IL-27 and TGFβ. While the exact molecular mechanism governing this differential induction of IL-10 in Th cells has not been explored, we speculate based on recent evidence that differential induction of transcription factors, such as Blimp-1 and c-maf might play an important role 34,35. Indeed, recent studies suggest that IL-27 mediated induction of IL-10 in Th1 cells is dependent on Blimp-1, while TGFβ mediated induction of IL-10 Tregs is independent of Blimp-1 34. Moreover, the effect of IL-27 and TGFβ blockade could also be indirect through an effect on APCs. Thus, a complex network of transcription factor induction could play a pivotal role in the differential requirement for expansion of IL-10^+^ CD4^+^ T cell subsets in active TB and should be investigated in the future. Co-inhibitory receptors, including CTLA-4 and PD-1, are known to play an important role in the regulation of the immune response in TB. While PD-1 is known to influence Th1 responses in active TB 36, CTLA-4 is known to impact the generic CD4^+^ T cell response to the pathogen 37. Our data reveal a very minimal role for both these receptors in the regulation of IL-10^+^ Th cells since they appear to significantly impact only the IL-10^+^ Th17 and aTreg subsets, although the smaller sample size used for this particular experiment might be a limiting factor.

In summary, IL-10 production by effector T cells during infectious and inflammatory responses is an important mechanism regulating the immune response. While the role of IL-10 in TB has been well-studied, the present study extends these to identify the cellular origins of the IL-10 and to elucidate the factors regulating these T cell subsets. Our data clearly highlight an important role for IL-10^+^ Th1 and aTreg cells in response in *M. tuberculosis* infection but also in the pathogenesis of tuberculous disease.

## Materials and Methods

### Study population

We studied a group of 54 individuals with pulmonary TB (PTB) and 20 individuals with latent TB (LTB). Individuals with pulmonary TB were diagnosed by positive solid cultures in Lowenstein–Jensen medium. Smear grades were determined by sputum microscopy and culture was graded as 0, 1^+^, 2^+^, and 3^+^ with zero being no bacteria in microscopy and 3^+^ the highest number of bacteria. The extent of chest disease was assessed by Chest X-ray and was classified as unilateral or bilateral disease for the purposes of this study. Individuals were diagnosed as having LTB on the basis of being positive in the Quantiferon-TB Gold in Tube (Cellestis) assay but having an absence of pulmonary symptoms concurrent with a normal chest radiograph. The two groups of individuals were age and sex matched. All subjects had been bacillus Calmette–Guerin (BCG) vaccinated at birth. All the individuals were HIV negative, not diabetic or suffering from autoimmune disorders and blood was collected prior to commencement of anti-TB treatment. This natural history study protocol was approved by the Institutional Review Board of the National Institute of Research in Tuberculosis and informed written consent was obtained from all participants.

### Antigens

Mycobacterial antigens—PPD (Statens Serum Institute, Copenhagen, Denmark), ESAT-6 and CFP—10 (both from NIAID TB antigen repository at BEI resources) were used as antigenic stimuli, and anti-CD3 antibody was used as positive control. Final concentrations were 10 μg/ml for PPD, ESAT-6 and CFP-10, and 5 μg/ml for anti-CD3.

### In vitro culture

Whole blood cell cultures were performed to determine the in vitro responses to antigens. Briefly, whole blood was diluted 1:1 with RPMI1640 medium supplemented with penicillin/streptomycin (100 U/100 mg/ml), L-glutamine (2 mM), and HEPES (10 mM; all from Invitrogen) and distributed in 12-well tissue culture plates (Costar). The cultures were then stimulated with ESAT-6, CFP-10, or anti-CD3 or with medium alone in the presence of CD49d/CD28 at 37°C for 18 h. Brefeldin A (10 µg/mL) was added after 12 h. After 18 h, centrifugation, washing, and red blood cell lysis was performed. The cells were fixed using cytofix/cytoperm buffer (BD Biosciences, San Jose, CA) and stored at −80°C. For neutralization experiments, whole blood was cultured in the presence of anti-IL-27 (5 μg/ml), anti-TGFβ (5 μg/ml) (R&D Systems, Minneapolis, MN), anti-PDL-1 (5 μg/ml) (eBiosciences, San Diego, CA), and anti CLTA-4 (5 μg/ml) (Ancell), or isotype control antibody (5 μg/ml) (R&D Systems) at 37°C for 6 h following which PPD was added and Brefeldin A (10 µg/ml) was added after 1 h. The cells were then cultured for a further 16 h.

### Intracellular cytokine staining

The cells were thawed, washed with cold PBS and permeabilized with 1× permeabilization buffer (Foxp3 staining buffer from eBiosciences). Cells were then stained with surface antibodies for 30 min and washed twice. The cells were then stained with intracellular antibodies and incubated overnight for 4°C. Antibodies used were as follows: T cell panel: CD3, CD4 and CD8; Th1 panel: IFNγ and T-bet; Th2 panel: IL-4 and GATA-3; Th9 panel: IL-9, Th17 panel: IL-17; Treg panel: CD25 and Foxp3; and IL-10. nTregs were defined as CD4^+^, CD25^+^, Foxp3^+^ and aTregs as CD4^+^, CD25-, Foxp3- expressing IL-10 alone. Eight-color flow cytometry was performed on a FACSCanto II flow cytometer with FACSDiva software, version 6 (Becton Dickinson, Franklin Lakes, NJ). The lymphocyte gating was set by forward and side scatter, and 100,000 lymphocytes events were acquired. Data were collected and analyzed using Flow Jo software (TreeStar, Ashland, OR). Frequencies following stimulation with antigens are depicted as net frequencies (with baseline values subtracted).

### Statistical analysis

Geometric mean was used as the measure of central tendency. Comparisons were made using either the Mann–Whitney test (unpaired comparisons) or the Wilcoxon signed rank test (paired comparisons). Comparisons were also made using one way Anova Post test for linear trends. All statistics were performed using GraphPad Prism version 5 for Windows (GraphPad Software, Inc.).

## Supporting Information

Figure S1 : Gating strategy and representative plots for IL-10 expressing CD4^+^ T cell subsets. (A) The gating strategy for CD4^+^ T cells from a representative individual. (B) A representative flow cytometry dot plot showing the expression patterns of IL-10^+^ IFNγ^+^, IL-10^+^ IL-4^+^, IL-10^+^ IL-9^+^, IL-10^+^ IL-17^+^ and IL-10^+^ Foxp3^+^ CD4^+^ T cells ex vivo and following stimulation with CFP-10 and anti-CD3 from a PTB individual. (C) A representative histogram and dot plot to show that IL-10^+^ IFNγ^+^ T cells were also T-bet^+^, while IL-10^+^ IL-4^+^ T cells were GATA-3^+^.

Figure S2 : Expansion of IL-10 expressing CD4^+^ T cell subsets in response to TB antigens and anti-CD3 in LTB. (A) Whole blood from LTB individuals (n=20) was stimulated with PPD, CFP-10, ESAT-6 or anti-CD3 for 24 and the frequencies of IL-10^+^ Th1 cells (IL-10^+^ IFNγ^+^ T-bet^+^); IL-10^+^ Th2 cells (IL-10^+^ IL-4^+^ GATA-3^+^); IL-10^+^ Th9 cells (IL-10^+^ IL-9^+^ IL-4^-^); IL-10^+^ Th17 cells (IL-10^+^ IL-17^+^ IFNγ^-^); IL-10^+^ nTregs (IL-10^+^ CD25^+^ Foxp3^+^) and IL-10^+^ aTregs (IL-10^+^ CD25^−^ Foxp3^−^) were estimated by flow cytometry. Results are shown as line diagrams with each line representing a single individual. *P* values were calculated using the Wilcoxon signed rank test.
