# Supplementary figures and images for: IL-27 and TGFβ mediated expansion of Th1 and adaptive regulatory T cells expressing IL-10 correlates with bacterial burden and disease severity in pulmonary tuberculosis
Source: Immun Inflamm Dis. 2015 Jun 18;3(3):289–99. doi: 10.1002/iid3.68 (PMC4578527; doi:10.1002/iid3.68)

Supplementary Figure 1A

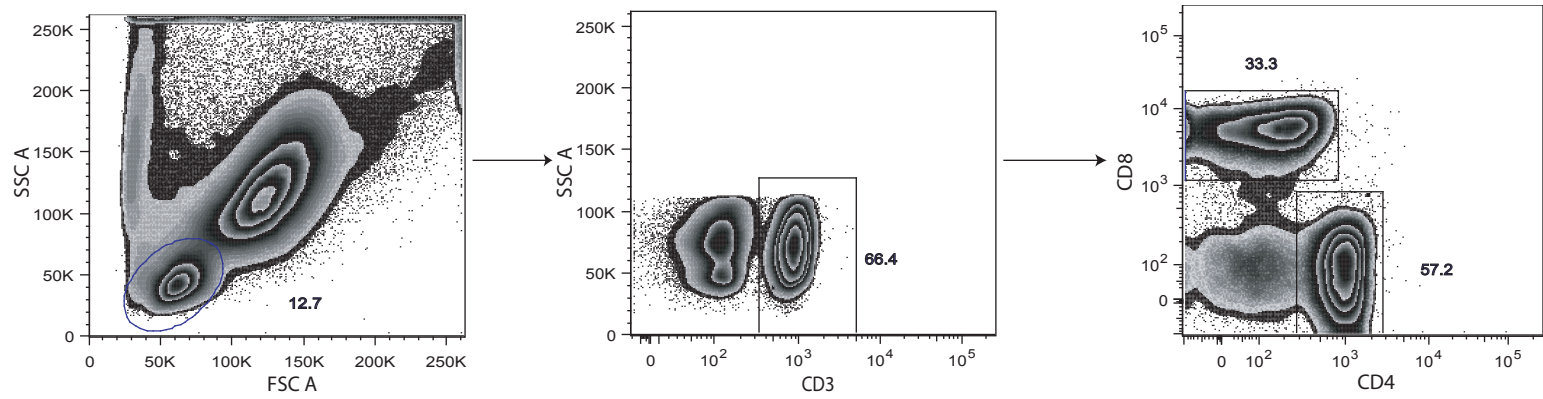

Supplementary Figure 1B

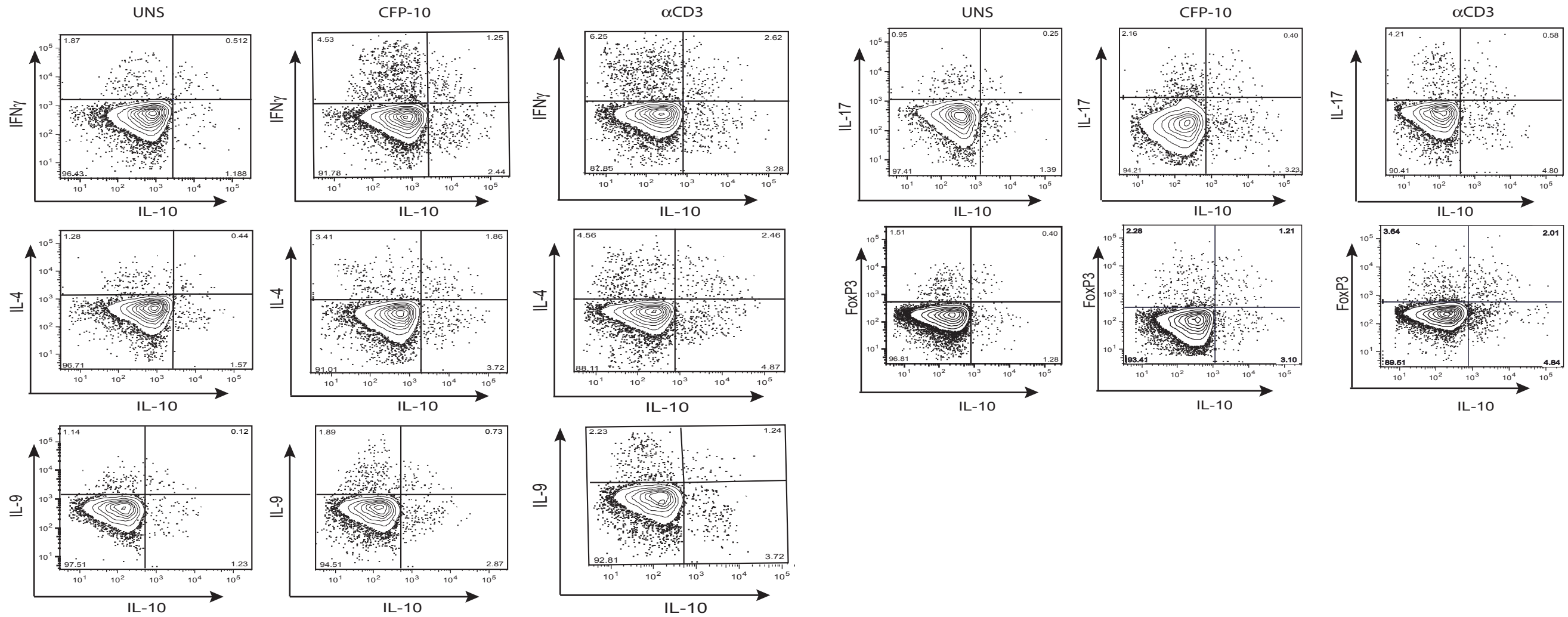

Supplementary Figure 1C

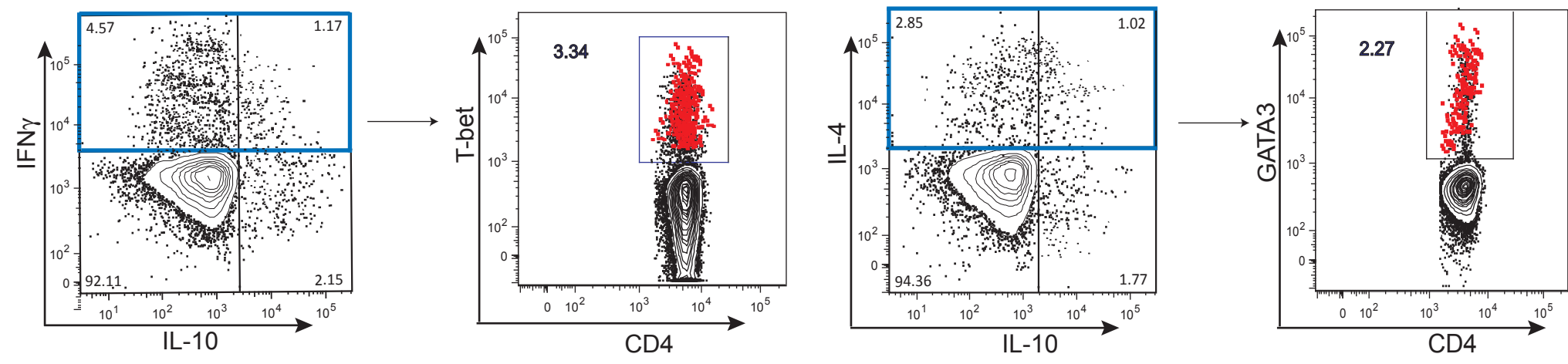

Supplement: Figure S1 — : Gating strategy and representative plots for IL-10 expressing CD4+ T cell subsets. (A) The gating strategy for CD4+ T cells from a representative individual. (B) A representative flow cytometry dot plot showing the expression patterns of IL-10+ IFNγ+, IL-10+ IL-4+, IL-10+ IL-9+, IL-10+ IL-17+ and IL-10+ Foxp3+ CD4+ T cells ex vivo and following stimulation with CFP-10 and anti-CD3 from a PTB individual. (C) A representative histogram and dot plot to show that IL-10+ IFNγ+ T cells were also T-bet+, while IL-10+ IL-4+ T cells were GATA-3+. [file iid30003-0289-sd1.pdf]
