# Supplementary material for: IL-27 and TGFβ mediated expansion of Th1 and adaptive regulatory T cells expressing IL-10 correlates with bacterial burden and disease severity in pulmonary tuberculosis
Source: Immun Inflamm Dis. 2015 Jun 18;3(3):289–99. doi: 10.1002/iid3.68 (PMC4578527; doi:10.1002/iid3.68)
Supplement: Figure S2 — : Expansion of IL-10 expressing CD4+ T cell subsets in response to TB antigens and anti-CD3 in LTB. (A) Whole blood from LTB individuals (n=20) was stimulated with PPD, CFP-10, ESAT-6 or anti-CD3 for 24 and the frequencies of IL-10+ Th1 cells (IL-10+ IFNγ+ T-bet+); IL-10+ Th2 cells (IL-10+ IL-4+ GATA-3+); IL-10+ Th9 cells (IL-10+ IL-9+ IL-4-); IL-10+ Th17 cells (IL-10+ IL-17+ IFNγ-); IL-10+ nTregs (IL-10+ CD25+ Foxp3+) and IL-10+ aTregs (IL-10+ CD25− Foxp3−) were estimated by flow cytometry. Results are shown as line diagrams with each line representing a single individual. P values were calculated using the Wilcoxon signed rank test. [file iid30003-0289-sd2.pdf]

Supplementary Figure 2

CD4+ Th1 ($Tbet^+IFN\gamma^+IL\text{-}10^+$)

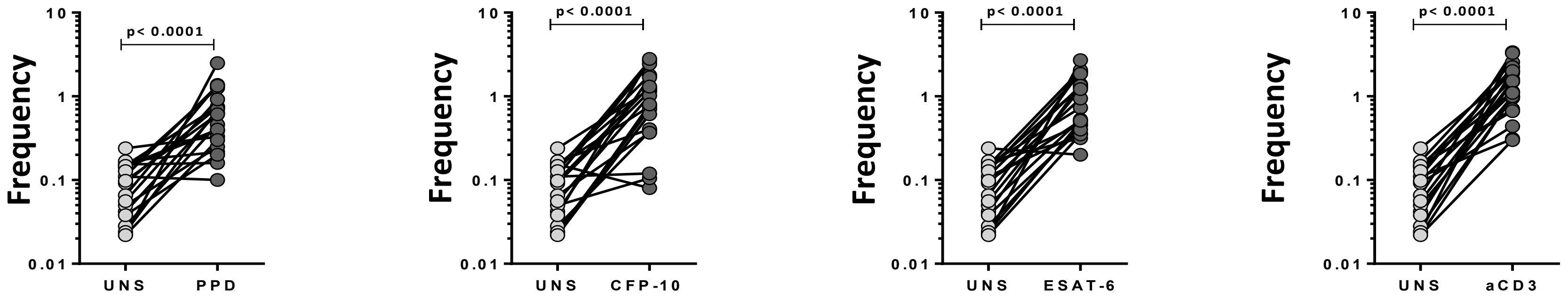

CD4+ Th2 ($GATA3^+IL\text{-}4^+IL\text{-}10^+$)

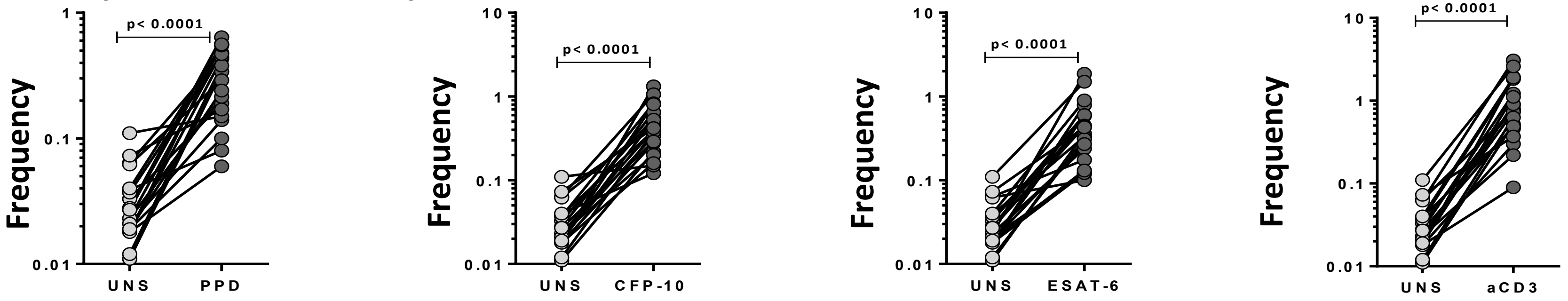

CD4+ Th17 ($IL\text{-}17^+IL\text{-}10^+$)

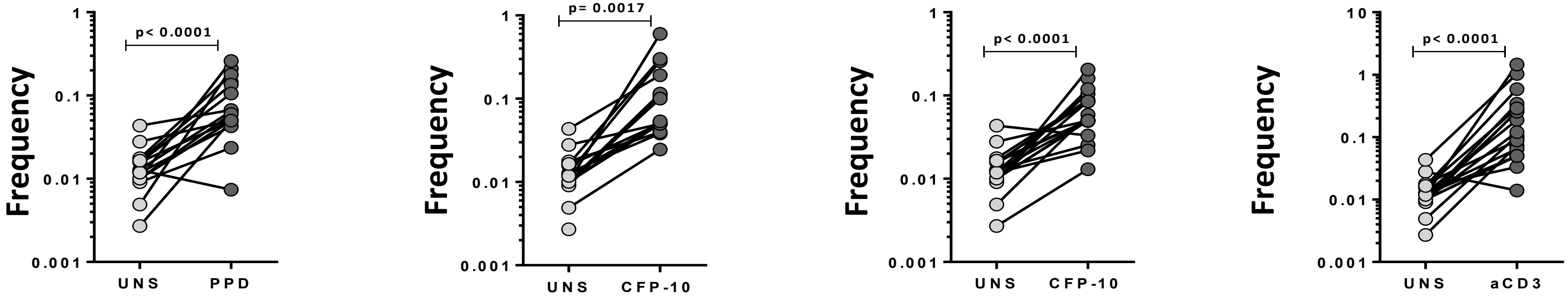

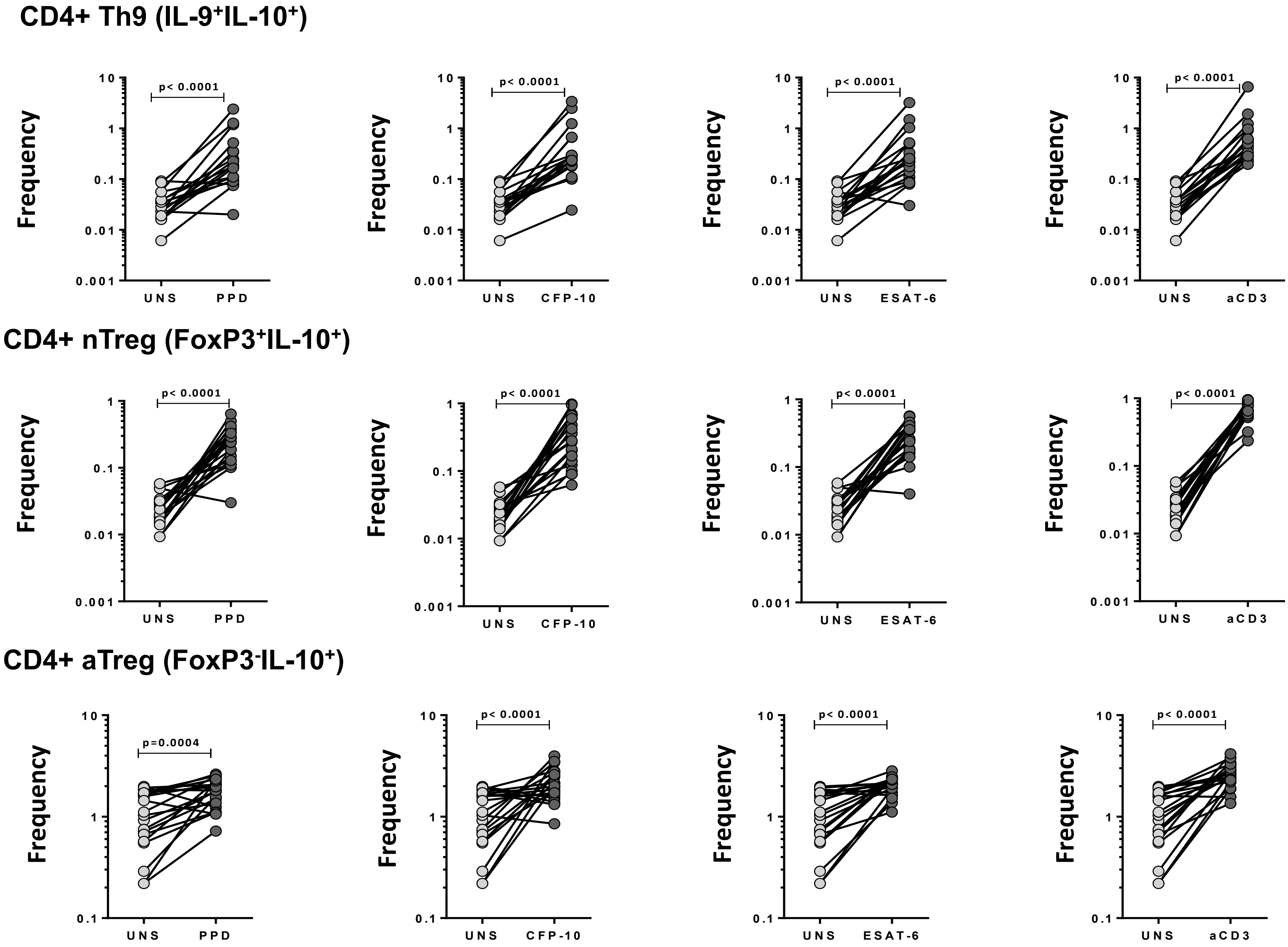
CD4+ Th9 (IL-9+IL-10+)
CD4+ nTreg (FoxP3+IL-10+)
CD4+ aTreg (FoxP3-IL-10+)
Frequency
UNS
PPD
CFP-10
ESAT-6
aCD3
p< 0.0001
p=0.0004
10
1
0.1
0.01
0.001
